# Towards a Miniaturized Photoacoustic Detector for the Infrared Spectroscopic Analysis of SO_2_F_2_ and Refrigerants

**DOI:** 10.3390/s23010180

**Published:** 2022-12-24

**Authors:** Hassan Yassine, Christian Weber, Nicolas Brugger, Jürgen Wöllenstein, Katrin Schmitt

**Affiliations:** 1 Department of Microsystems Engineering, University of Freiburg, 79110 Freiburg, Germany; 2 Fraunhofer Institute for Physical Measurement Techniques IPM, 79110 Freiburg, Germany

**Keywords:** photoacoustic spectroscopy, infrared, sulfuryl fluoride (SO_2_F_2_), FTIR, refrigerants

## Abstract

Sulfuryl fluoride (SO_2_F_2_) is a toxic and potent greenhouse gas that is currently widely used as a fumigant insecticide in houses, food, and shipping containers. Though it poses a major hazard to humans, its detection is still carried out manually and only on a random basis. In this paper, we present a two-chamber photoacoustic approach for continuous SO_2_F_2_ sensing. Because of the high toxicity of SO_2_F_2_, the concept is to use a non-toxic substituent gas with similar absorption characteristics in the photoacoustic detector chamber, i.e., to measure SO_2_F_2_ indirectly. The refrigerants R227ea, R125, R134a, and propene were identified as possible substituents using a Fourier-transform infrared (FTIR) spectroscopic analysis. The resulting infrared spectra were used to simulate the sensitivity of the substituents of a photoacoustic sensor to SO_2_F_2_ in different concentration ranges and at different optical path lengths. The simulations showed that R227ea has the highest sensitivity to SO_2_F_2_ among the substituents and is therefore a promising substituent detector gas. Simulations concerning the possible cross-sensitivity of the photoacoustic detectors to H_2_O and CO_2_ were also performed. These results are the first step towards the development of a miniaturized, sensitive, and cost-effective photoacoustic sensor system for SO_2_F_2_.

## 1. Introduction

The detection of sulfuryl fluoride (SO_2_F_2_) has gained increasing interest in recent years since it is widely used as a fumigant insecticide in houses, food, and shipping containers [1]. SO_2_F_2_ is neurotoxic and therefore poses a major hazard to humans, e.g., to inhabitants of treated houses or workers in the logistics industry, with an occupational exposure limit (OEL) of 5 ppm and an immediate danger for life and health starting from 200 ppm [2]. Since the adoption of the Montreal Protocol in 1987, in which the use of methyl bromide as a fumigant was phased out because of its harm to the ozone layer, SO_2_F_2_ has largely replaced methyl bromide, and the amount used drastically increased to more than 2000 metric tons per year [3,4]. Apart from its toxicity, SO_2_F_2_ is a potent greenhouse gas with a global warming potential of 4780 and an atmospheric lifetime of 36 years [5,6]. The main degradation pathway in the environment is hydrolysis, e.g., in oceans and wetlands [6,7,8].

Due to the high necessity of SO_2_F_2_ measurement to minimize its use and therefore release into the environment, a wealth of different sensors and sensor principles have recently been developed and have been reported in the literature [9,10,11,12,13,14,15,16]. Apart from fumigation, SO_2_F_2_ detection is often employed in the failure monitoring of fully enclosed insulated gas switching equipment (gas-insulated switchgear, GIS) because SO_2_F_2_ is a decomposition product of SF_6_-filled GIS. Such sensors must be able to monitor SO_2_F_2_ at very low concentrations (low ppm to ppb range) to safely warn below the OEL of 5 ppm. Most of the proposed sensors involve transition metals [9,13] or semiconductors [10,11], which detect a chemical reaction of SO_2_F_2_ with the respective material. Such sensors have the advantage of being very sensitive and compact, yet they often suffer from cross-sensitivities to other gases and ambient parameter variations, such as temperature and humidity. Several studies on metal/semiconductor-based sensors relied on density functional theory (DFT) simulations of chemical reactions with SO_2_F_2_ and took changes in the ambient conditions into account [12,13,14,15,16].

Alternatively, gases can often also be measured very well optically via their characteristic absorption in the infrared, as they have distinct absorption bands in the spectral range between 3 µm and 16 µm. Simple non-dispersive IR (NDIR) gas sensors work with a few fixed spectral channels and pyroelectric detectors. To minimize influences from interfering gases or drift effects from the light source, an additional reference channel is usually used for compensation, which measures in an absorption-free wavelength range. The wavelengths and spectral bandwidths are selected using thin-film interference filters, which are each optimized for the wavelength of the absorption band of the gas to be measured [17,18]. Naik et al. [19] reported an NDIR-based sensor for SO_2_F_2_ using a spectral absorption around 6.64 µm for the concentration range of 500–30,000 ppm. For higher sensitivity and resolution, SO_2_F_2_ can also be detected using tunable laser absorption spectroscopy (TDLAS) with suitable light sources and optical long-path cells, as proposed in [20,21]. Yao et al. [20] employed on interband cascade laser (ICL) at a 3619 nm central wavelength, combined with a 5.33 long-path cell to reach a detection limit of ~4 ppm. Zhang et al. [21] conducted a simulation study on the comparison of an ICL at 3619 nm and a quantum cascade laser (QCL) at 6653 nm, either using TDLAS or photoacoustic detection to reach a sub-ppm detection limit: 0.34 ppm for the ICL and 0.66 ppm for the QCL. Photoacoustics is another highly selective infrared spectroscopic measurement technique and is widely used to measure IR-active gases at low concentrations, from high-precision industrial process monitoring to trace gas monitoring [22,23,24]. The operating modes of photoacoustic systems can be divided into resonant and non-resonant. Resonant cells are mainly used in trace gas monitoring, which requires a very high level of sensitivity. With lasers as light sources, resonant photoacoustic systems can achieve sensitivities in the ppb range [25,26]. However, resonant systems are comparatively complex and expensive. Recently, different resonant photoacoustic systems have been introduced for SO_2_F_2_ sensing [27,28,29,30], achieving such low detection limits. However, such systems are laboratory-based due to their size and complexity. In contrast, non-resonant systems can be much smaller and less complex, making them suitable for use outside the laboratory. Broadband infrared sources such as filament emitters or planar thermal emitters are often employed. An advantage of broadband emitters is that all absorption lines of the target gas contribute to the signal, which allows the optical path length to be reduced. A prominent application example is room climate monitoring, in which the CO_2_ concentration is evaluated as a parameter for air quality [31].

**Figure 1:**
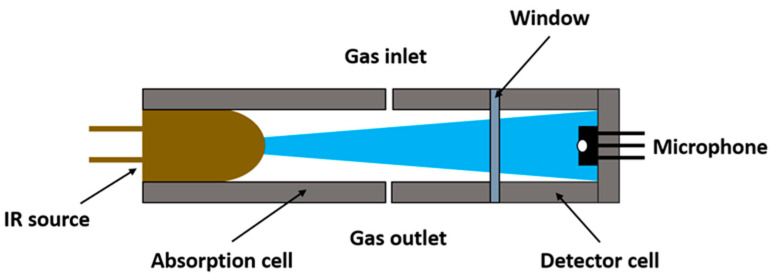
Scheme of a two-chamber photoacoustic sensor. The light of a modulated source passes through the absorption cell with an unknown target gas concentration. The detector cell with the microphone is hermetically sealed and filled with the target gas or a substituent. The detector signal decreases with an increasing target gas concentration in the absorption cell.

Therefore, we propose a non-resonant, two-chamber photoacoustic approach to meet the requirements of size, cost, and complexity reduction to obtain a sensor for continuous SO_2_F_2_ sensing in field applications, e.g., freight container monitoring. The scheme of a two-chamber photoacoustic sensor is shown in Figure 1. Our core concept is to use a non-toxic substituent gas with similar absorption characteristics in the photoacoustic detector chamber for indirect, but still selective, SO_2_F_2_ detection. Fourier-transform infrared (FTIR) spectroscopy was used to obtain high-resolution spectra of the possible substituents 1,1,1,2,3,3,3-heptafluoropropane (R227ea), 1,1,1,2,2-pentafluoroethane (R125), 1,1,1,2-tetrafluoroethane (R134a), and propene. The resulting infrared spectra were used to simulate the sensitivity of the substituents of a photoacoustic sensor to SO_2_F_2_ in different concentration ranges and at different optical path lengths.

## 2. Materials and Methods

### 2.1. Measurement Setup

To estimate the sensitivity of the hermetically sealed photoacoustic detector cells to SO_2_F_2_, simulations were performed, requiring high-resolution spectra of the target gas, SO_2_F_2_, and the possible substituents. For this purpose, the transmission measurements of SO_2_F_2_ and the substituent gases were determined using an FTIR spectrometer (Vertex v80, Bruker, Billerica, MA, USA). The decadic absorption coefficients were calculated from the resulting spectra.

**Figure 2:**
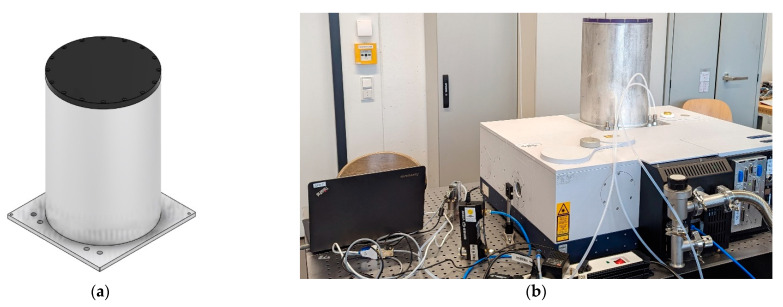
(**a**) CAD model of the designed lid. An aluminum cylinder (250 mm × 15 mm × 360 mm) with a polycarbonate plate (250 mm × 15 mm) was fixed with screws to a rectangular aluminum plate (282 mm × 293 mm × 10 mm). (**b**) Measurement setup with the lid fixed to the FTIR spectrometer with the long-path gas cell inside. The gas inlet was connected to one of the bulkhead fittings on the outside of the lid, and a second bulkhead fitting was connected to the gas exhaust. The inlet and the outlet of the long-path gas cell were connected to the respective bulkhead fittings on the inside of the lid.

The measurement setup consisted of the FTIR spectrometer combined with a 10 m long-path gas cell (Pike Technologies, Madison, WI, USA), mass flow controllers (Bronkhorst, The Netherlands), and a lid (Figure 2a). The lid was needed to evacuate the sample compartment of the spectrometer to avoid absorption from atmospheric gases. However, the long-path gas cell was higher than the sample compartment, so the sample compartment could not be evacuated with the standard lid of the spectrometer. A lid with corresponding dimensions, as seen in Figure 2a, was constructed and designed. An aluminum cylinder (250 mm × 15 mm × 360 mm) with a polycarbonate plate (250 mm × 15 mm) was fixed through screws to a rectangular aluminum plate (282 mm × 293 mm × 10 mm). Four holes for straight bulkhead fittings were inserted in the bottom plate. The gas inlet was connected to one of the bulkhead fittings on the outside of the lid, and a second bulkhead fitting was connected to the gas exhaust (see Figure 2b). The inlet and the outlet of the long-path gas cell were connected to the respective bulkhead fittings on the inside of the lid. In case the sample compartment was intended to be flushed with nitrogen gas (N_2_), the third bulkhead fitting was connected to the N_2_ source and the fourth one was connected to the gas exhaust. For evacuating, these two were closed so that the entire measurement setup remained vacuum tight.

Before adjusting different concentrations of each gas and recording the resulting spectra with the FTIR spectrometer, the measurement parameters, such as the mirror velocity, resolution, etc., were set. For all measurements, consistent parameters were defined. A mercury–cadmium–telluride (MCT) photo detector cooled with liquid N_2_ was used to achieve high sensitivity. Table 1 summarizes all parameters.

**Figure 3:**
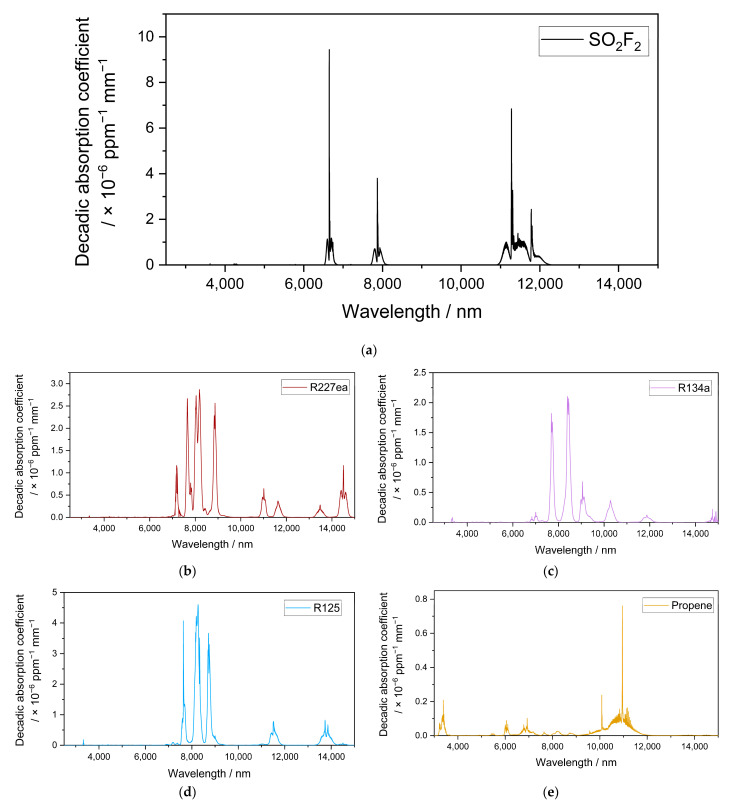
(**a**) Decadic absorption coefficient spectrum of SO_2_F_2_ between 2500 and 15,000 nm and the decadic adsorption coefficient spectra of the possible substituents R227ea (**b**); R134a (**c**) [32]; R125 (**d**) and propene (**e**) in standard conditions.

The transmission spectra were recorded after setting different gas concentrations, starting from the gas cylinder concentration down to 5–15 ppm. Certified gas bottles were obtained from Westfalen, Germany (1000 ppm SO_2_F_2_ in N_2_), and TEGA, Germany (refrigerants), and used as received. At high gas concentrations, the strong absorption bands were already saturated, while weak absorption bands were not saturated and were clearly measured. On the other hand, at low gas concentrations, the strong absorption bands were clearly measured, while the weak ones were not. The decadic absorption coefficients were calculated from the transmission spectrum at each concentration, and the calculated spectra were merged. The resulting spectrum then contained both the weak and strong absorption bands with the desired resolution (Figure 3).

### 2.2. Simulations

**Figure 4:**
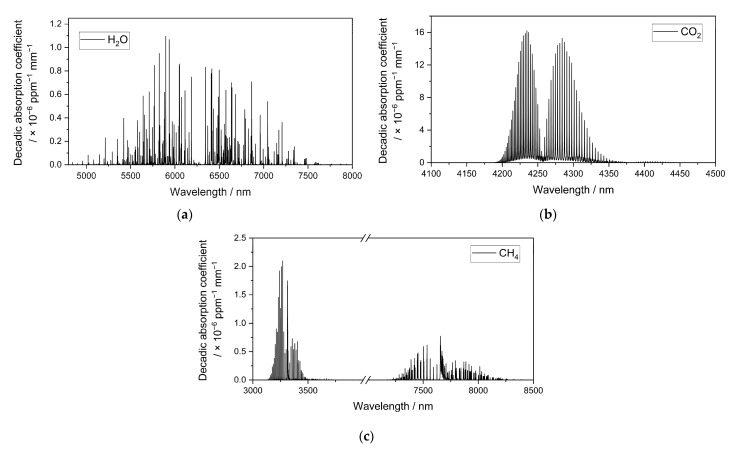
Decadic absorption coefficient spectra of (**a**) H_2_O between 4500 and 8000 nm, (**b**) CO_2_ between 4100 nm and 4500 nm, and (**c**) CH_4_ between 3000 nm and 8500 nm in standard conditions, which were calculated and plotted using the HITRAN database [34].

The sensitivity of the photoacoustic detectors filled with the substituent pure gas to the target gas, SO_2_F_2_, was simulated. This was performed using a script written in JavaScript, similar to that in [32]. The script calculated the integral absorbed power in the detector cell filled with a definite pure gas (100 Vol.-%). By varying the concentration of the target gas in the absorption path at a defined optical path length, the integral absorbed power in the detector decreased. This change was defined as the sensitivity. Furthermore, a suitable window material was selected for the hermetic sealing of the detectors. Silicon has an average transmission of about ~50% in the infrared region [33]. It is easy to obtain and can easily be sawed into desired small detector windows. For this purpose, the transmission measurements of a single-side-polished (SSP) Si wafer and a double-side-polished (DSP) Si wafer were determined using the FTIR spectrometer (Figure 4).

The numerical calculations were performed for each substituent gas using the decadic absorption coefficients calculated from the FTIR measurements and by considering the IR transmission of the DSP Si wafer. Both the detector gas and the optical path length were varied.

(1) $$I_{Emitter}\left(\lambda \right)=\frac{dP_{Emitter}}{d\lambda }=\frac{2\pi \cdot h\cdot c^{2}}{\lambda^{5}}\cdot \frac{A}{\exp\left(\frac{h\cdot c}{k_{B}\cdot \lambda \cdot T}\right)-1}$$

(2) $$A\left(\lambda \right)=1-T\left(\lambda \right)=1-10^{-\alpha \cdot \left(\lambda \right)\cdot l\cdot c},$$

(3) $$P_{Abs}=\int_{\lambda_{1}}^{\lambda_{2}}I_{Emitter}\left(\lambda \right)\cdot A_{Detector}\left(\lambda \right)\cdot T_{Window}\left(\lambda \right)\cdot T_{Abs.\;cell}\left(\lambda \right)\;d\lambda ,$$

where *I* is the emitted spectral power of the emitter, *P_Emitter_* is the optical power of the emitter, *λ* is the wavelength, *h* is Planck’s constant, *c* is the speed of light, *A* is the active emitter area of the emitter, *k_b_* is Stefan–Boltzmann’s constant, *T* is the temperature, *α* is the decadic absorption coefficient of the gas, *T_window_* and *T_Abs.cell_* are the transmission through the window and the absorption cell, respectively, *A_Detector_* is the absorption in the detector, and *P_Abs_* is the integral absorbed power in the detector cell. Using these Equations, the emitted spectral power of the infrared emitter, with an active emitter area 2.2 mm × 2.2 mm, and the integral absorbed power in the detector were calculated [35,36]. The length of the detector chamber was fixed to 1.5 mm. The sensitivity of the detectors filled with SO_2_F_2_ and the refrigerants (100 Vol.-%) to 0–10,000 ppm SO_2_F_2_ at an optical path length of 50 mm was simulated. Similarly, simulations for the measurement range of 0–50 ppm SO_2_F_2_ with an optical path length of 1.6 m were conducted. The cross-sensitivity to atmospheric gases such as H_2_O (0–4 Vol.-%) and CO_2_ (0–2000 ppm) were also investigated for both optical path lengths.

## 3. Results and Discussion

### 3.1. Infrared Spectroscopic Characteristics of SO_2_F_2_ and Refrigerants

The resulting infrared spectra of SO_2_F_2_ and the possible substituents are plotted in Figure 3. SO_2_F_2_ had three main strong absorption bands in the wavelength range between 6500 and 6800 nm, 7700 and 8100 nm, and 10,500 and 12,200 nm (Figure 3a).

As seen in Figure 3b and Table 2, R227ea had absorption bands that overlapped with SO_2_F_2_ between 7700 and 8100 nm, 10,900 and 11,250 nm, and 11,400 and 11,800 nm. R134a had bands that overlapped with the target gas between 6550 and 6800 nm, 7700 and 8050 nm, and 11,000 and 12,200 nm [32]. R125 had bands that overlapped with the target gas between 7700 and 8100 nm and 10,900 and 12,000 nm. Propene had weaker absorption compared with the other refrigerants but also had bands that overlapped with SO_2_F_2_ between 6500 and 6800 nm, 7700 and 7800 nm, 7900 and 8100 nm, and 10,950 and 12,000 nm.

Figure 4 shows the decadic absorption spectra of the possibly interfering atmospheric gases H_2_O, CO_2_, and CH_4_, which were calculated and plotted using the HITRAN database [34].

### 3.2. Simulation of the Sensitivity of the Photoacoustic Detectors

**Figure 5:**
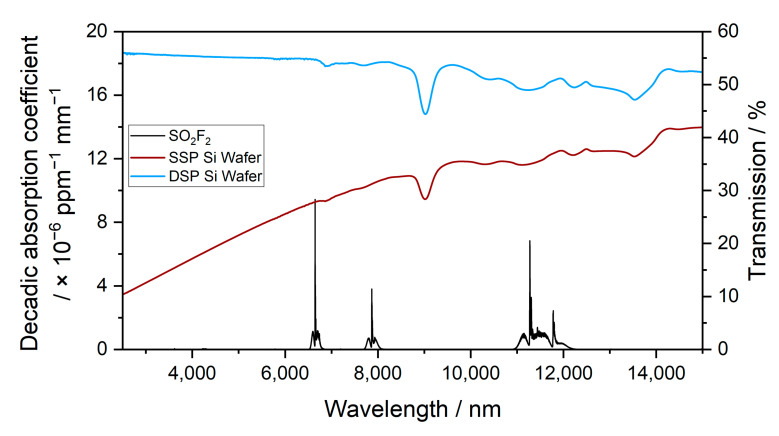
Decadic absorption coefficient spectrum of SO_2_F_2_ and the infrared transmission through 500 µm of SSP Si and 500 µm of DSP Si between 2500 and 15,000 nm.

The infrared transmission through 500 µm of SSP Si and 500 µm of DSP Si and the spectrum of SO_2_F_2_ in the wavelength range between 2500 and 15,000 nm are shown in Figure 5. The infrared transmission through DSP Si was higher than the transmission though SSP Si. The average infrared transmission through DSP Si was about 52 % in the wavelength ranges where SO_2_F_2_ absorbs.

**Figure 6:**
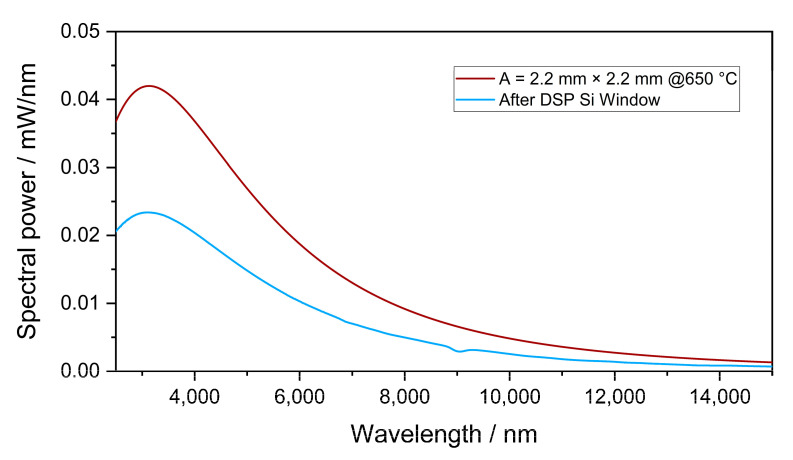
Simulated emitted spectral power of the infrared emitter with an emitter temperature of 650 °C and an active area of 2.2 mm × 2.2 mm and the transmitted spectral power through DSP Si.

Figure 6 shows the emitted spectral power of the infrared source simulated using Equation 1 with an emitter temperature of 650 °C as well as the transmitted spectral power through DSP Si. According to these simulations, the minimum spectral power transmitted into the detector was about 0.006 mW/nm at wavelengths between 7800 and 8100 nm, and it dropped to about one third at wavelengths between 10,500 and 12,200 nm. Nevertheless, the transmitted power was sufficient for the planned application.

**Figure 7:**
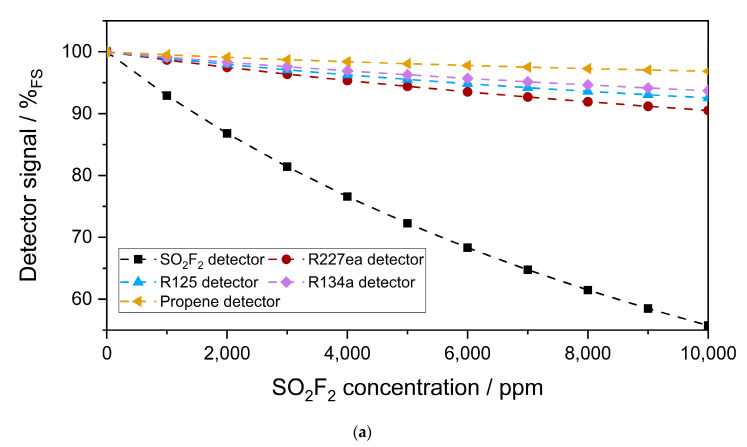

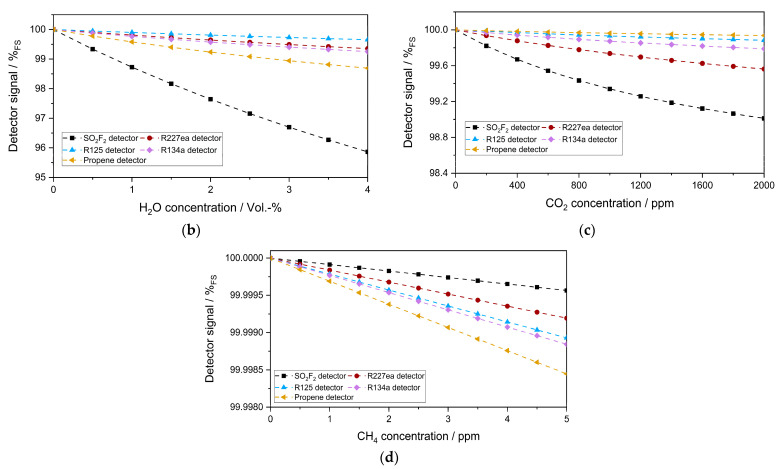
(**a**) Simulated sensitivity of the photoacoustic detectors to 0–10,000 ppm SO_2_F_2_ in the absorption cell (50 mm length) and the cross-sensitivity to ambient H_2_O (0–4 Vol.-%) (**b**) and ambient CO_2_ (0–2000 ppm) (**c**) as well as ambient CH_4_ (0–5 ppm) (**d**).

The simulated sensitivity of each photoacoustic detector to 0–10,000 ppm SO_2_F_2_ in the absorption cell (50 mm length) is plotted in Figure 7a. As expected, the highest sensitivity was obtained with the SO_2_F_2_ detector. Among the substituents, R227ea showed the highest sensitivity to SO_2_F_2_. Although R125 absorbed stronger than R227ea, R134a, and propene between 10,500 and 12,200 nm, the absorbance of R227ea between 7700 and 8100 nm was higher than those of R134a, R125, and propene, and the spectral power transmitted through the detector between 7700 and 8100 nm was three times higher than that between 10,500 and 12,200 nm. For this reason, the R227ea detector was more sensitive to SO_2_F_2_ than the other detectors. The propene detector showed the lowest sensitivity to SO_2_F_2_, which can be explained by its weak infrared absorption compared to the other substituents in the considered wavelength range. Moreover, the relative signal change of the SO_2_F_2_ detector versus the SO_2_F_2_ concentration change in the absorption cell was not linear, while that of the refrigerant detectors was.

Figure 7b–d show the cross-sensitivity of the photoacoustic detectors to 0–4 Vol.-% H_2_O, 0–2000 ppm CO_2_, and 0–5 ppm CH_4_, respectively. The highest cross-sensitivity was obtained with SO_2_F_2_ as the detector gas. The relative signal change of a SO_2_F_2_ detector to ambient 0–4 Vol.-% H_2_O in the absorption cell was 10× lower than that to 0–10,000 ppm SO_2_F_2_. This is because the absorption bands of SO_2_F_2_ overlap with those of H_2_O, especially at 6500–6800 nm. The cross-sensitivity of the SO_2_F_2_ detector to ambient CO_2_ (0–2000 ppm) was 4× lower than that to ambient H_2_O. The lowest cross-sensitivity to ambient H_2_O was obtained with the R125 detector, while that to CO_2_ was obtained with propene. The R227ea detector showed a maximum relative signal change of 0.5 % to CO_2_ and H_2_O. The cross-sensitivity of the photoacoustic detectors to 0–5 ppm CH_4_, which is in the range of the relevant background concentration in the atmosphere, was negligible. However, the problem of the cross-sensitivity of the photoacoustic detectors to CO_2_ and H_2_O must be taken into account when calibrating the sensors and can be solved by additionally integrating humidity and CO_2_ sensors into a final sensor system or by integrating a humidity sensor and an IR-blocking filter.

**Figure 8:**
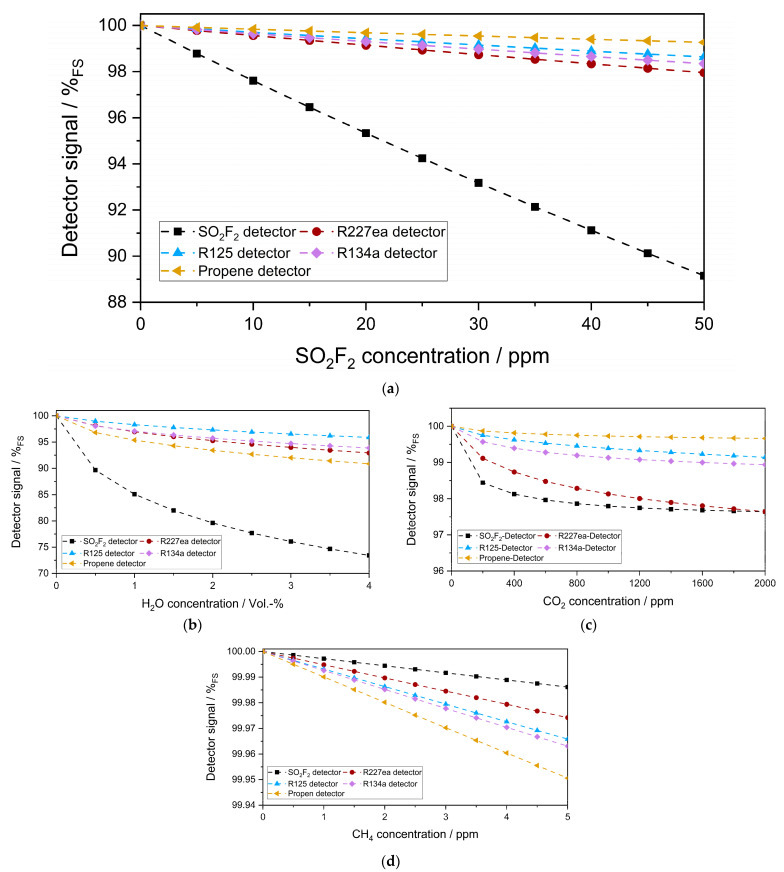
(**a**) Simulated sensitivity of the photoacoustic detectors to 0–50 ppm SO_2_F_2_ in the absorption cell (1.6 m) and the cross-sensitivity to ambient H_2_O (0–4 Vol.-%) (**b**) ambient CO_2_ (0–2000 ppm) (**c**) and ambient CH_4_ (0–5 ppm) (**d**).

Figure 8a shows the simulated sensitivity of each photoacoustic detector to 0–50 ppm SO_2_F_2_ in a long-path absorption cell (1.6 m optical path length). In these simulations, only the concentrations of SO_2_F_2_ and the optical path length were changed. Everything else was kept as in the previous simulations. Therefore, R227ea still showed the highest sensitivity to SO_2_F_2_ among the substituents. Moreover, the relative signal change of the SO_2_F_2_ detector as a function of the SO_2_F_2_ concentration change in the long-path absorption cell became linear, like that of the refrigerant detectors. The cross-sensitivity of the photoacoustic detectors to 0–4 Vol.-% H_2_O and 0–2000 ppm CO_2_ also became more critical in this case (Figure 8b,c), while the cross-sensitivity to 0–5 ppm CH_4_ was still low and negligible (Figure 8d).

## 4. Conclusions

In this article, an investigation of a new photoacoustic detector approach for the measurement of SO_2_F_2_ is presented. It is based on the concept of filling a non-toxic substituent gas that has absorption bands that overlap with the target gas into a photoacoustic detector chamber to measure SO_2_F_2_ indirectly. The refrigerants R227ea, R125, R134a, and propene were selected as possible substituents. An infrared spectroscopic analysis of SO_2_F_2_ and the refrigerants was performed using an FTIR spectrometer. The resulting infrared spectra were used as a basis for simulating the sensitivity of the detector filled with the substituents to SO_2_F_2_ in different concentrations and at different optical path lengths. In addition, the cross-sensitivity of the detectors filled with the substituents to ambient CO_2_ and H_2_O was simulated. The simulations revealed that R227ea had the highest sensitivity to SO_2_F_2_ among the substituents and could be used as a detector gas. Detection limits below 50 ppm and 1.5 ppm could be reached with a 50 mm absorption cell and a 1.6 m long-path absorption cell, respectively, with a thermal broadband emitter and a compact setup. The simulation results also showed the cross-sensitivity of the photoacoustic detectors to ambient gases such as H_2_O and CO_2_, which have to be taken into account in experimental studies. Based on these results, photoacoustic sensors for the measurement of SO_2_F_2_ will be designed, built, and experimentally characterized in the future.

## Figures and Tables

**Table 1:** Parameters used for the transmission measurements with the FTIR spectrometer (Vertex 80v, Bruker).

Detector Type	LN-MCT Photoconductor
Resolution	0.08 cm^−1^
Mirror velocity	80 kHz
Acquisition mode	Single-sided, forward–backward
Phase correction method	Mertz
Apodization function	Three-term Blackman–Harris window

**Table 2:** List of the substituent gases with the wavelength ranges of absorption that overlapped with SO_2_F_2_.

Substituent	Wavelength Range of Absorption Overlapping with SO_2_F_2_ (nm)
R227ea	7700–8100
	10,900–11,250
	11,400–11,800
R134a [32]	6550–6800
	7700–8050 11,000–12,200
R125	7700–8100
	10,900–12,000
Propene	6500–6800
	7700–7800 7900–8100 10,950–12,000

## Data Availability

The data presented in this study are available in [this article].

## References

[1] Derrick M.R., Burgess H.D., Baker M.T., Binnie N.E. (1990). Sulfuryl fluoride (Vikane): A review of its use as a fumigant. J. Am. Inst. Conserv..

[2] (2019). NIOSH Pocket Guide to Chemical Hazards. “#0581”.

[3] Veryser C., Demaerel J., Bieliūnas V., Gilles P., De Borggraeve W.M. (2017). Ex situ generation of sulfuryl fluoride for the synthesis of aryl fluorosulfates. Org. Lett..

[4] Gressent A., Rigby M., Ganesan A.L., Prinn R.G., Manning A.J., Mühle J., Salameh P.K., Krummel P.B., Fraser P.J., Steele L.P. (2021). Growing atmospheric emissions of sulfuryl fluoride. J. Geophys. Res. Atmos..

[5] Papadimitriou V.C., Portmann R.W., Fahey D.W., Mühle J., Weiss R.F., Burkholder J.B. (2008). Experimental and theoretical study of the atmospheric chemistry and global warming potential of SO_2_F_2_. J. Phys. Chem. A.

[6] Sulbaek Andersen M.P., Blake D.R., Rowland F.S., Hurley M.D., Wallington T.J. (2009). Atmospheric chemistry of sulfuryl fluoride: Reaction with OH radicals, Cl atoms and O_3_, atmospheric lifetime, IR spectrum, and global warming potential. Environ. Sci. Technol..

[7] Mühle J., Huang J., Weiss R.F., Prinn R.G., Miller B.R., Salameh P.K., Simmonds P.G. (2009). Sulfuryl fluoride in the global atmosphere. J. Geophys. Res. Atmos..

[8] Roshni V., Harikumar V.S. (2021). Fluoride contamination in wetlands of Kuttanad, India: Predisposing edaphic factors. Eurasian Soil Sci..

[9] Qian H., Deng J., Xie Z., Pan Z., Zhang J., Zhou H. (2020). Adsorption and gas sensing properties of the Pt_3_-MoSe_2_ monolayer to SOF_2_ and SO_2_F_2_. ACS Omega.

[10] Liu H., Zhou Q., Zhang Q., Hong C., Xu L., Jin L., Chen W. (2017). Synthesis, characterization and enhanced sensing properties of a NiO/ZnO p–n junctions sensor for the SF_6_ decomposition byproducts SO_2_, SO_2_F_2_, and SOF_2_. Sensors.

[11] Li L., Din S.U., ul Haq M., Tang N., Zhang M., Rahman N., Zhu L. (2021). Room temperature monitoring of SF_6_ decomposition byproduct SO_2_F_2_ based on TiO_2_/NiSO_4_ composite nanofibers. Nanotechnology.

[12] Gui Y., Wang Y., Duan S., Tang C., Zhou Q., Xu L., Zhang X. (2019). Ab initio study of SOF_2_ and SO_2_F_2_ adsorption on Co-MoS_2_. ACS Omega.

[13] Liu Z., Gui Y., Xu L., Chen X. (2022). Adsorption and sensing performances of transition metal (Ag, Pd, Pt, Rh, and Ru) modified WSe_2_ monolayer upon SF_6_ decomposition gases (SOF_2_ and SO_2_F_2_). Appl. Surf. Sci..

[14] Gui X., Zhou Q., Peng S., Xu L., Zeng W. (2020). Adsorption behavior of Rh-doped MoS_2_ monolayer towards SO_2_, SOF_2_, SO_2_F_2_ based on DFT study. Phys. E Low-Dimens. Syst. Nanostruct..

[15] Fá A.G., Faccio R., López-Corral I. (2021). Detection of SOF_2_ and SO_2_F_2_ through aluminium nitride nanosheets: A DFT study. Appl. Surf. Sci..

[16] Huang H., Yu Y., Zhang M. (2020). Analysis of adsorption properties of SF_6_ decomposed gases (SOF_2_, SO_2_F_2_, SF_4_, CF_4_, and HF) on Fe-doped SWCNT: A DFT study. Appl. Surf. Sci..

[17] Dinh T.V., Choi I.Y., Son Y.S., Kim J.C. (2016). A review on non-dispersive infrared gas sensors: Improvement of sensor detection limit and interference correction. Sens. Actuators B.

[18] Bogue R. (2015). Detecting gases with light: A review of optical gas sensor technologies. Sens. Rev..

[19] Naik R.C., Shroff R.D. NDIR based SO_2_F_2_ detector for fumigation monitoring. Proceedings of the 9th International Conference on Controlled Atmosphere and Fumigation in Stored Products.

[20] Yao Q., Yan X., He S., Qi R., Li X., Zeng X.-Z., Wang X., Zhang S., Zi J., Yue Y. (2021). Detection of SO_2_F_2_ concentration of SF_6_ decomposition product in GIS gas chamber based on ICL-TDLAS. Adv. Sens. Syst. Appl. XI.

[21] Zhang S., Qiang Y. (2022). Study on the suitable intermediate infrared spectrum optical detection applied to SO_2_F_2_ and SOF_2_. Int. Workshop Adv. Algorithms Control. Eng..

[22] Bozóki Z., Pogany A., Szabo G. (2011). Photoacoustic instruments for practical applications: Present, potentials, and future challenges. Appl. Spectrosc. Rev..

[23] Hodgkinson J., Tatam R.P. (2012). Optical gas sensing: A review. Meas. Sci. Technol..

[24] West G.A., Barrett J.J., Siebert D.R., Reddy K.V. (1983). Photoacoustic spectroscopy. Rev. Sci. Instrum..

[25] Schilt S., Thévenaz L., Niklès M., Emmenegger L., Hüglin C. (2004). Ammonia monitoring at trace level using photoacoustic spectroscopy in industrial and environmental applications. Spectrochim. Acta Part A Mol. Biomol. Spectrosc..

[26] Sigrist M.W. (1995). Trace gas monitoring by laser-photoacoustic spectroscopy. Infrared Phys. Technol..

[27] Bian C., Dai F., Cheng J., Chen X., Gan Q., Zhang Z., Tan T., Yang B., Wang C., Cui G. (2021). Detection of SF_6_ decomposition components SO_2_F_2_ and SOF_2_ based on mid-infrared laser photoacoustic spectroscopy. Int. Symp. High Volt. Eng..

[28] Minini K.M.S., Bueno S.C.E., da Silva M.G., Sthel M.S., Vargas H., Angster J., Miklós A. (2017). Quantum cascade laser-based photoacoustic sulfuryl fluoride sensing. Appl. Phys. B.

[29] Zhang Y., Wang M., Yu P., Liu Z. (2022). Optical Gas-Cell Dynamic Adsorption in a Photoacoustic Spectroscopy-Based SOF_2_ and SO_2_F_2_ Gas Sensor. Sensors.

[30] Zhang Y., Wang M., Yu P., Liu Z. (2022). Optical gas sensing of sub-ppm SO_2_F_2_ and SOF_2_ from SF_6_ decomposition based on photoacoustic spectroscopy. IET Optoelectron..

[31] Huber J., Ambs A., Rademacher S., Wöllenstein J. (2014). A selective, miniaturized, low-cost detection element for a photoacoustic CO_2_ sensor for room climate monitoring. Proc. Eng..

[32] El-Safoury M., Weber C., Kiesewetter O., Hespos Y., Eberhardt A., Schmitt K., Wöllenstein J. (2020). Miniaturized photoacoustic detection of organofluorine-based refrigerants. J. Sens. Sens. Sys..

[33] Kuznetsova T.I., Lebedev V.S. (2004). Transmission of visible and near-infrared radiation through a near-field silicon probe. Phys. Rev. B.

[34] Gordon I.E., Rothman L.S., Hill C., Kochanov R.V., Tan Y., Bernath P.F., Birk M., Boudon V., Campargue A., Chance K.V. (2017). The HITRAN2016 Molecular Spectroscopic Database. J. Quant. Spectrosc. Radiat. Transf..

[35] Demtröder W. (2018). Experimentalphysik 2: Elektrizität und Optik.

[36] Baehr H.D., Stephan K. (1994). Wärme-Und Stoffübertragung.

